# Hospital and Institutional News

**Published:** 1920-07-31

**Authors:** 


### HOSPITAL AND INSTITUTIONAL NEWS.

### SIR A. DEMPSEY, M.D.

We regret to announce the death of Sir Alexander Dempsey, M.D., J.P., which recently occurred at his residence, "Coldagh," Somerton Road, Fort William, Belfast. He was a prominent member of the medical profession in Ireland, and his reputation as a skilled and successful practitioner was by no means confined to the city of Belfast, with which his career is generally associated. He was gynæcologist and consulting physician to the Mater Infirmorum Hospital, and to this institution he devoted much of his untiring zeal and energy. In the year 1911 the honour of knighthood was conferred upon him in recognition both of his public services, which were many, and his distinctions as a member of the medical profession.

### PROFESSOR GUYON.

It is with the greatest regret that we learn of the death of Professor Felix Guyon in Paris. He was formerly President of the Academie of Sciences and the Academy of Medicine, and his work as the Chief of the Hôpital Necker in connection with genito-urinary disease is world-famous. His early studies were made in Nantes and Paris, and he combined the true skill and dexterity of the first-class surgeon with an invaluable aptitude for progressive research. He was the author of many well-known treatises, and as early as 1876 he published his pioneer experiences with Lister's teachings applied to practical surgery. He was a conscientious and thorough teacher whose life-work has undoubtedly greatly advanced the progress of medicine.

### WHAT ABOUT THE RED CROSS APPEAL?

Sir Napier Burnett's hospital survey was, we understood, to be quickly followed by the issue of an appeal for the provincial hospitals, many of which, he has demonstrated, are in a perilous financial position, and must envy the London institutions in that they have a Central Fund able and willing to make an emergency grant. Carefully made schemes for money raising are not prepared in a few hours or days, and we suspect that, concurrently with the preparation of the survey, wise heads have been busy weaving a wide and systematic appeal for funds. It is not generally appreciated in London that the provincial hospitals are in quite a different position as to patients' payments. In most districts the working population give freely and regularly, and would not understand a system which would be quite applicable to London, where most of the hospitals receive patients from no particular district. On the other hand, the provincial hospitals have the advantage over London, not only in the working-class support, but because of the irresistible appeal to the rich residents of the neighbourhood, almost every hospital having its distinct giving public.

### CANCER RESEARCH.

The annual meeting of the Imperial Cancer Research Fund was held on July 22 at Queen's Square, Bloomsbury, under the presidency of the Duke of Bedford. Various announcements were made describing new channels of valuable investigational work opening up from the earlier research which our contributor has described in our article published this week (see page 451). Particularly noteworthy was Dr. Murray's method of observing and testing the malignancy of animal tumours by transplanting a portion to another part of the same animal's body —*i.e.*, producing artificial metastases. Also attempts are being made to differentiate between benign and malignant growths through the agency of chemical tests. The possibility of specific action by certain chemical agents or by the typical cancer cell is certainly fascinating therapeutically considered, but at present it appears as though most of the differences in chemical activity are directly due to the *extra rapidity* of growth metabolism in the cells of a neoplasm, rather than to any specific chemical property of the malignant cell.

### AMENDING THE LUNACY LAWS.

Recently a deputation representing the Mental Hospitals' Association waited upon Lord Astor at the Ministry of Health with the object of urging the Government to concede increased grants enabling the authorities to carry on their work with greater efficiency. The point made for the Association was that the existing lunacy laws in protecting society and the liberty of the subject allowed insufficient scope for the treatment and cure of patients. On this occasion, as when Sir James Crichton Browne ably described the position some weeks ago (see The Hospital, May 29, 1920), the urgent necessity for provision of treatment in the early and curable stages of mental disorder was brought forward and the fact explained that a small Bill amending the Lunacy Act could make it possible without certification, to achieve this end. We are glad to note that Lord Astor promised to consider sympathetically this suggestion and said that legislation would be forthcoming in some at least of the lines indicated. In this connection, too, we would draw attention to our correspondent's article on page 453 of our present issue.

### AMERICA AND BRITAIN.

As a momento of the co-operation between the Americans and British in surgical work during the war a number of surgeons in this country have decided to make a presentation to the American College of Surgeons (which includes Canada as well as U.S.A.), and with this end in view a silver gilt mace has been made for them by the well-known worker in metal Mr. Omar Ramsden. A point of interest in design comes with the fact that the head is modelled on the lines of a surgeon's mortar, which was dug up during trench cutting in Salonika, and the decoration is brought to include both the maple leaf and the emblematic American eagle. The roll of subscribers will be engraved later under the badges of the Army Medical Corps of both countries. The inscription at present reads, "From the consulting surgeons of the British Armies to the American

College of Surgeons in memory of the mutual work and good fellowship in the Great War." The mace has been placed in the Exhibition of Industrial Art at 217 Knightsbridge, London, where it will be on view for about a month.

### THE LISTER MEMORIAL FUND.

At the recent meeting of the General Committee of the Lister Memorial Fund proposals were detailed for carrying out the scheme adopted in 1912 whereby an international memorial fund should be established. In *précis* they were as follows:—(*a*) That £500 out of the general fund, together with a bronze medal, be awarded every three years, irrespective of nationality, in recognition of distinguished contributions to surgical science, the recipient being required to give an address in London under the auspices of the Royal College of Surgeons of England; (*b*) that the award be made by two members nominated by the Royal Society, two by the Royal College of Surgeons (England), one by the Royal College of Surgeons (Ireland) or by Edinburgh University, and one by Glasgow University; (*c*) surplus income, after providing for erection of a monument and after defraying administrative expense, be either devoted to grants for furtherance of surgical science, or invested to increase the capital of the fund. To carry out the scheme requires establishment of permanent machinery, and the Royal College of Surgeons of England will probably become the trustees and administrators of the fund, and thus proceed to carry out its objects. The subscription list remains open, and the Hon. Treasurer, Sir Watson Cheyne, Bt., will received donations at the Royal Society, Burlington House, London, W. 1.

### THE RED CROSS £250,000.

King Edward's Hospital Fund, who are the almoners for the British Red Cross Society in respect of the grant of £250,000 for schemes of extension and improvement at London hospitals, have wisely given on the principle, generally, of few gifts and good ones. As will be seen by the account of the meeting of the Fund to be found on page 455, less than fifty hospitals participate, and seven of these absorb half the money between them. The hospitals chosen are not all of them usually included in the list of those receiving grants from the Fund, notably St. Bartholomew's, granted £24,000 for the nurses' home, and the Woolwich War Memorial Hospital, awarded a grant of £25,000. Viscount Finlay, who moved the adoption of the report and awards, said that the munificent gift of the Joint Committee could not have come at a time when it was more needed, as many schemes had been deferred during the war and had become urgent. This is very true, but the pity is that the cost of building will in many instances prohibit the immediate use of the money. Another point is that the grants are not expected to cover the entire cost of the proposed works, and the struggle for funds for maintenance will interfere with raising the remainder of the cost of buildings. But in such cases the Committees will be able to go on with renewed energy, having the knowledge that such a comfortable nucleus has been formed.

### INCOME-RAISING DEVICE IN THE ANTIPODES.

Our correspondence column this week contains a letter from an Australian medical man, making a suggestion for income-raising which is new to this country, although it has been carried out in several hospitals in the Antipodes. Briefly, it concerns a licence for visitors to be allowed to see their friends at other than the regular hours, or, rather, on certain "extra visiting hours" suited to the convenience of the hospital. To obtain the privilege a sixpenny ticket has to be bought, and it is stated that the sale of these tickets brings in from £300 to £500 a year for every hundred beds. At first sight the system seems to bear hardly on the poor patient, or, rather, the patient with poor friends, but it was found that the Australian public welcomed the innovation. It would be interesting to learn how the proposal is regarded by those in the home hospitals who have most to do with the regulation of visitors and visiting hours.

### THE BRITISH HOSPITALS' ASSOCIATION AND PATIENTS' PAYMENTS.

Viscount Hambleden has issued a circular letter informing the hospitals that the London Committee of the British Hospitals' Association have at an influentially attended meeting resolved that in their opinion, having regard to the financial position, the time has arrived when the principle of requiring patients to contribute towards the cost of their maintenance should be recommended with a view to its general adoption in London.

### HOSPITALS AND EXCESS PROFITS DUTY.

We are glad to note every attempt made by London hospitals to enlist the regular support of firms in their neighbourhood, though the attempt is not so general as one could wish. Mr. Panter, Secretary of the Great Northern Central Hospital, however, took advantage of the concession dating from July 16 under the excess profits duty to send a statement to the leading firms, in which he said the Finance Act now allows "firms who pay excess profits duty to make a deduction in respect of contributions to hospitals from the amount of the tax payable. The actual allowance is on the basis of an amount not exceeding 5 per cent. of excess profits as calculated for the purposes of the excess profits duty before adjustments for increased or decreased capital, and before making any deduction under this statute, and not exceeding 20 per cent. of the contributions to hospitals. The provision goes beyond any contribution which would be admissible as a deduction from the profits for the purposes of the excess profits duty. This concession (the Chancellor stated) has been made in the hope that it will encourage the trading houses to recognise the needs of the communities in which their successful businesses are conducted." The claim of the hospital is then urged, and firms are invited to be generous in return for the concession made to them. We hope that they will, and that this avenue of approach to them will lead to more methodical support, which is badly needed, especially in London.

### SURREY'S WAR FUNDS.

The War Fund raised by the Lord Lieutenant of Surrey has now been wound up, and the remaining money will be allocated as follows:—£3,000 to be divided equally among the Surrey Convalescent Home for Men at Seaford, the Convalescent Home for Women at Bognor, and the Children's Convalescent Home at Bognor, for the maintenance of beds for ex-soldiers, their wives and dependants in the administrative county of Surrey and Croydon; £4,000 to Earl Haig's Officers' Association Fund, for the benefit of Surrey and London officers, their families and dependants; £1,000 each to the Surrey Division of the Comrades of the Great War, and the National Federation of Discharged Soldiers and Sailors, for the relief of discharged men out of employment; £1,000 to the Soldiers and Sailors' Help Society (Surrey branch), to assist ex-Service men; £2,000 to the Surrey Red Cross Society for a scheme of curative posts in Surrey for ex-Service men and dependants; £3,750 between the Reigate and Redhill Hospital, the Royal Richmond Hospital, and the Sutton Hospital (War Memorial) for building fund or other capital expenditure. The Royal Surrey County Hospital receives £1,250 for building or other capital expenditure as well as the residue of the fund, after outstanding liabilities have been settled.

### SHOULD MIDWIVES HAVE SALARIES OR FEES?

According to a report of the Heston and Isleworth District Council the increase in births is "taxing the present arrangements to their utmost capacity." The medical officer of health says there is urgent need for one or more midwives in the district, for he is informed there is no midwife in Heston. He suggests that the Council might copy the example of the Middlesex County Council, which has appointed several salaried midwives in connection with the maternity and child-welfare scheme. The County then receives the fees—an arrangement which seems to work well. If the salaries are sufficient there is much to be said in their favour, for the midwife needs a secure income, more perhaps than any other nurse.

### NEW CONVALESCENT HOME FOR LIVERPOOL.

A handsome gift has been made to the Liverpool Hospital Saturday Fund with the presentation, by Mrs. Hugh Rathbone, of Crofton Hall, Aigburth, as a convalescent home for women. During the war the hall was used as a military hospital, and, therefore, is well equipped, beside possessing agreeable grounds. Crofton Hall is not the first gift from the Rathbone family to Liverpool, and is an instance of the hereditary interest which hospital work excites in the families which become acquainted with it. The Saturday Funds of the Midlands and the North are rich in institutions of this kind, which are supported with admirable public spirit by the members.

### THE DILEMMA AT GRAVESEND.

The Gravesend Hospital is badly in debt and at a loss to know where to look for fresh support. Although the workers of the neighbourhood and the pilots are known to support the hospital, we would ask Mr. Chapman, the Secretary, if a methodical attempt has been made to organise a subscription fund from the employers of the district. If not, the note on "Hospitals and Excess Profits duty" provides an instance how they can be immediately approached, and employers' funds should include the names of the principal retailers, a point not always remembered in areas where factories, in the current sense, are few. Since the Chairman is strongly opposed to maintenance charges, for reasons readily to be guessed, an employers' subscription fund seems a reasonable alternative.

### A ROMAN CATHOLIC HOSPITAL IN BOMBAY.

Under the patronage of the Archbishop, Roman Catholics of Bombay have launched an appeal for the establishment in the city of a Catholic General Hospital. A number of leading Catholics, including the Hon. Major Fernandes, are to act as managing trustees.

### CHILD WELFARE IN BOMBAY.

Mrs. Rossent, who is the superintendent of the Bombay Infant Welfare Centres, is an associate of the Royal Sanitary Institute, London, holder of a hygiene diploma, and is a fully-trained nurse and has been nursing and teaching in India for seven years. She is now on her way home to study the latest infant welfare methods. She will return in the autumn to Bombay. The committee is meanwhile getting new premises and providing a number of trained Indian workers, so that all may be in readiness for the superintendent.

### ASYLUM HOSPITALITY AT CARDIFF.

Dr. Edwin Goodall, medical superintendent of Cardiff Mental Hospital, has submitted a big extension scheme, which will be necessary if the Cardiff Corporation shall obtain the further powers which it has sought. Provisionally the scheme provides for 380 further patients at a cost of £500,000. In response to a request from Dr. Evans Hoyle, Secretary of the Committee in charge of accommodation for visitors to the conference of the British Association at Cardiff, it was decided to offer him accommodation for visitors at the Mental Hospital. The offer is one of four dormitories with thirty-five beds apiece, together with sitting-rooms in which breakfast and cold supper will be served. Dr. Goodall states that the junior nursing staff has agreed to do the necessary work. The charge for this accommodation will be decided later.

### MENTAL HOSPITAL CHARGES.

The maintenance charges at mental hospitals rise like those at other hospitals, but less is heard of them because they are not paid by voluntary funds. At Glamorgan County Mental Hospital the charge for outside patients is 40s. 3d.; at Newport

Mental Hospital it is now 35s. At Cardiff Mental Hospital the charge has been lately raised from 32s. 11d. to 35s., and a further advance is expected. Before the war, we understand, the charge was under 10s.

### A HOSPITAL SATURDAY PRESIDENT'S BEQUESTS.

Leicester charities benefit to the extent of £3,500 under the will of the late Mr. J. Gipson Clarke, J.P., who succeeded Sir Edward Wood in the Presidency of the Leicester and County Saturday Hospital Society. Legacies of £1,000 are bequeathed to the Leicester Royal Infirmary, £5,000 to the Leicester and County Saturday Hospital Society, £500 each to the Leicester Guild of the Crippled and the Ebenezer Chapel, St. Peter's Lane, Leicester, and £200 each to the Leicester Blind Institution, the Leicester Wycliffe Society for Helping the Blind, the Leicester Surgical Aid Society, the Poor Boys' and Girls' Summer Camp, and the Institution for Diseases of the Skin. Mr. Clarke was a generous contributor to all worthy local causes.

### NESTON COTTAGE HOSPITAL.

The opening of a Cottage Hospital at Neston, in Cheshire, is the result of the example set by a Red Cross Hospital during the war. A house known as "Dee View" was bought, and paid for, and the new institution has been opened by Sir Norman Hill. Mr. M. Glover is the secretary, and Mr. Larden Williams the treasurer. Apart from a private ward and the staff's quarters there are three wards, two with three beds, and women's ward with five. The colliers have already promised regular support, and an income of £600 a year is needed.

### A METHODICAL PLAN IN BELFAST.

In Belfast the organisation of subscriptions from employers and workpeople has already made progress. Last year the firms alone gave £8,477, and the men also a large sum. In view of the rise in prices, however, Mr. James Mackie, chairman of the financial aid committee, is trying to get both donors to agree to double their gifts. He and Mr. H. Benington, the hospital's treasurer, have been busy personally visiting the heads of the various firms, and the confession which we report elsewhere from the head of a Sheffield firm shows that this is the right method. If most of those asked agree, the men's subscriptions will amount to £20,000 and the employees to £15,000, an increase almost enough to meet the current cost of maintenance.

### THE SALARIES OF SECRETARIES.

The Board of Governors of Stockport Infirmary has unanimously decided to increase the salary of the Secretary-Superintendent to £600 per annum. The Chairman spoke highly of the work of Mr. Edwin J. Pearce; the improvement in the ordinary income for the past half-year, which had increased by £2,335, and was largely due to his efforts. Mr. Pearce was formerly Secretary of the Royal Hampshire County Hospital, Winchester. Many improvements are being made at Stockport. The domestic offices are being enlarged, which include a new dining-room for the nurses; and the nurses' home is also being extended. Besides these, a new hot-water system, boiler-house, and electric lift are being installed. The total cost of those alterations is £24,000. Messrs. Daniel Eadie & Co., of Stockport, are responsible for the building, and Messrs. Ashwell & Nesbit, of Leicester, are the engineers. The amount of work and responsibility involved make a revision of the Superintendent's salary timely, and it is perhaps because these posts are few that their holders have had to wait so long for an adequate salary.

### MORE INSURANCE STAMPS.

The Unemployment Insurance Bill, which was the subject of an article in our issue of June 19, page 299, passed its third reading in the House of Commons on July 19, and is intended to come into operation on October 1 next. As non-manual workers earning up to £250 a year are included, a large number of cards and stamps will be required for institutional workers. The Bill appears to have few friends; it has been "praised with faint damns," as the Babu said, by the Government, and is cordially disliked by the Labour party, but it looks like inevitably becoming law, and, in the case of hospitals, will give a great deal of work with little benefit to the employee. *Apropos de bottes*, our attention has been specially drawn to the fact that the National Health Scheme now includes non-manual workers earning up to £250. Hospital managers have often, we suspect, regarded the operation of this Act, as it applies to institutional staffs, with the telescope to the blind eye; almost more than anywhere else the benefits in respect of hospital workers are illusory, for they have always, with or without insurance, obtained an equivalent to the benefits. We would like to see the facilities of contracting out much more attainable than they are at present.

### THIS WEEK'S DRUG MARKET.

The downward tendency of prices is not so pronounced at the moment, but business continues to be almost inactive, and signs of improvement are not in sight. In many cases prices are merely nominal, and it is probable that considerable concessions would have to be made to tempt buyers. In some instances it is quite improbable that prices will recover, in view of the fact that the output has increased and stocks have accumulated during the protracted lull in trade. Although there are no noteworthy declines in quotations for synthetic drugs, these quotations do not in all cases represent the figures at which business of any importance could be done. Cocaine hydrochloride is in better supply, and is offered at lower rates. There has been some improvement in the demand for menthol, and the price has firmed up a little. Japanese refined camphor is also in rather better request. Cod-liver oil still hangs fire. More business has been done in citric acid at lower rates. The best advice that can be given to buyers is to continue to hold off as long as possible, keeping, of course, an eye on the markets.

